# Variant Allele Frequency Analysis of Circulating Tumor DNA as a Promising Tool in Assessing the Effectiveness of Treatment in Non-Small Cell Lung Carcinoma Patients

**DOI:** 10.3390/cancers16040782

**Published:** 2024-02-14

**Authors:** Natalia Galant, Marcin Nicoś, Barbara Kuźnar-Kamińska, Paweł Krawczyk

**Affiliations:** 1 Department of Pneumonology, Oncology and Allergology, Medical University of Lublin, 20-059 Lublin, Poland; 2 Department of Pulmonology, Allergology and Respiratory Oncology, Poznan University of Medical Sciences, 61-710 Poznan, Poland; kaminska@ump.edu.pl

**Keywords:** NSCLC, liquid biopsy, circulating free DNA, circulating tumor DNA, variant allele frequency, personalized treatment

## Abstract

**Simple Summary:**

The non-invasive characteristic of liquid biopsy enables an increase in the potential of VAF analysis in monitoring tumor progression, remission, and recurrence during or after treatment. Moreover, the use of VAF analysis appears to be beneficial in making treatment decisions. Several studies have been performed on patients with NSCLC to evaluate the possibility of VAF usage. However, several issues require better understanding and standardization before VAF testing can be implemented in clinical practice. In this review, we discuss the difficulties in the application of ctDNA VAF analysis in clinical routine, discussing the diagnostic and methodological challenges in VAF measurement in liquid biopsy. We highlight the possible applications of VAF-based measurement in the monitoring of personalized treatment in patients with NSCLC who are under consideration in clinical trials.

**Abstract:**

Despite the different possible paths of treatment, lung cancer remains one of the leading causes of death in oncological patients. New tools guiding the therapeutic process are under scientific investigation, and one of the promising indicators of the effectiveness of therapy in patients with NSCLC is variant allele frequency (VAF) analysis. VAF is a metric characterized as the measurement of the specific variant allele proportion within a genomic locus, and it can be determined using methods based on NGS or PCR. It can be assessed using not only tissue samples but also ctDNA (circulating tumor DNA) isolated from liquid biopsy. The non-invasive characteristic of liquid biopsy enables a more frequent collection of material and increases the potential of VAF analysis in monitoring therapy. Several studies have been performed on patients with NSCLC to evaluate the possibility of VAF usage. The research carried out so far demonstrates that the evaluation of VAF dynamics may be useful in monitoring tumor progression, remission, and recurrence during or after treatment. Moreover, the use of VAF analysis appears to be beneficial in making treatment decisions. However, several issues require better understanding and standardization before VAF testing can be implemented in clinical practice. In this review, we discuss the difficulties in the application of ctDNA VAF analysis in clinical routine, discussing the diagnostic and methodological challenges in VAF measurement in liquid biopsy. We highlight the possible applications of VAF-based measurements that are under consideration in clinical trials in the monitoring of personalized treatments for patients with NSCLC.

## 1. Introduction

Lung cancer is one of the leading causes of death in oncological patients worldwide. It is histologically classified as non-small cell lung carcinoma (NSCLC) or small cell lung carcinoma (SCLC). NSCLC occurs more frequently and represents 85% of all lung cancer cases. It is also divided into three main subtypes: adenocarcinoma (40%), squamous cell carcinoma (25–30%), and large cell carcinoma (5–10%). Despite the many possible therapeutic strategies that can be applied to patients with NSCLC, the responses to them are still very diverse [1,2,3,4].

To enable the selection of beneficial therapy to patients, new biomarkers with potential clinical applications are still being investigated. Circulating tumor DNA (ctDNA), among other biomarkers, is attracting much attention from researchers [5]. The significance of ctDNA analysis in patients with NSCLC has increased in recent years. In contrast to tumor tissue genotyping, ctDNA isolation from a liquid biopsy is non-invasive and may be performed at different time points; thus, patients can be monitored over the entire duration of therapy without the risk of biopsy-related side effects. Besides the possibility of more frequent examination, liquid biopsy enables the genetic testing of patients at a high risk of NSCLC when access to tissue material is limited [5,6]. Furthermore, ctDNA analysis also has the potential to become a marker for the detection and monitoring of minimal residual disease (MRD) [7,8,9].

The recent breakthrough in next-generation sequencing (NGS), and its implementations into clinical routines, has provided a large variety of measuring metrics that increase the sensitivity of liquid biopsy testing [10,11]. Variant allele frequency (VAF) in ctDNA is being considered one of the markers with prospective clinical utility [12]. The possibility of its usage to assess the effectiveness of therapy in patients with NSCLC is being evaluated [13]. Research results give hope that VAF monitoring may provide information about response to treatment and patient prognosis, and help in developing optimal therapy. Furthermore, VAF changes seem to occur quickly, even before radiological evidence of response is noticeable. However, it should be taken into account that the clinical usage of VAF requires more broad-based research [14].

Considering the increasing interest in searching for measurable parameters in liquid biopsy, in this review, we discuss the differentiation of cancer and non-cancer DNA fractions in liquid biopsy. Furthermore, we indicate the diagnostic and methodological challenges in VAF measurements in liquid biopsy. Finally, we highlight the possible applications of VAF-based measurement in the monitoring of personalized treatments for NSCLC that are already available in research or under consideration in clinical trials.

## 2. cfDNA

**Figure 1:**
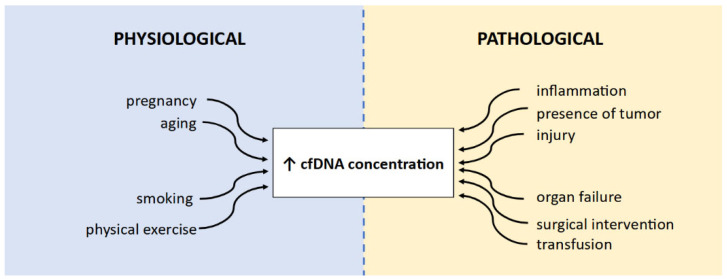
A summary of causes affecting cfDNA concentration increase. Based on [20,21].

Circulating free DNA (cfDNA) fragments are double-stranded DNA fragments circulating in body fluids, such as peripheral blood, cerebrospinal fluid (CSF), and urine. cfDNA fragments are short (below 200–220 base pairs, usually 167 bp), and their half-life in circulation ranges from 5 to 150 min. In healthy individuals, cfDNA is normally at low concentrations and enters body fluids mostly via apoptosis, neutrophil extracellular traps (NETs), and erythroblast enucleation [15,16,17,18]. Moreover, it was indicated that smaller, average, and larger-than-average cfDNA fragments may be products of necrosis [19]. cfDNA concentrations may be influenced by many factors, such as age or smoking status, and, in general, are higher in older patients compared to younger ones and in smokers compared to non-smokers [20]. It has also been indicated that cfDNA levels may be increased physiologically, for example, after physical exercise. High cfDNA concentrations may also be affected by pathological processes, such as inflammation or cancer. A summary of the causes for increases in cfDNA concentrations is presented in Figure 1. The fraction of cfDNA released by cancer cells is called circulating tumor DNA (ctDNA) [18,21]. While in oncological patients, the terms cfDNA and ctDNA are commonly used interchangeably, it is important to understand the differences between them.

### 2.1. ctDNA

ctDNA is a fraction of cfDNA released into the bloodstream by primary or metastatic tumor cells. Cancerous ctDNA and physiological cfDNA can be distinguished by a genetic background typical of tumors (mutations, rearrangements, or copy number alterations in selected genes) or by the abnormal methylation of gene promoters [22,23]. Furthermore, the important difference between ctDNA and cfDNA is their length, ctDNA can either be smaller or larger than cfDNA. Large ctDNA fragments (above 10,000 bp) may be released due to tumor cell necrosis [18,22]. However, of greatest interest to researchers are fragments with a length similar to cfDNA or shorter, as the selection of shorter fragments improves the ctDNA/cfDNA ratio. Moreover, as studies have proven, cfDNA fragments carrying mutant alleles are often shorter compared to those with wild-type alleles; thus, an analysis of shorter fragments may be beneficial during mutant allele frequency evaluation [24,25,26]. Additionally, the possibility of analyzing fragments with a length exceeding 10,000 bp is significantly limited due to methodological issues [27].

In oncological patients, the concentration of ctDNA may vary depending on the cancer type, stage of disease (especially tumor size), and presence of local or distant metastases [18,28,29]. A study performed on a group of 640 patients showed that ctDNA was detected more frequently (>75%) in patients with advanced pancreatic, colorectal, gastroesophageal, hepatocellular, bladder, ovarian, breast, and head and neck cancers or melanoma, while ctDNA was detected less often (<50%) in patients suffering from primary brain, prostate, thyroid, and renal cancers [30]. It was also found that ctDNA detection is positively related to cancer stage. Furthermore, the concentration of ctDNA is higher in patients with metastatic cancers compared to groups with localized disease [30,31]. Another study, including a group of 88 NSCLC patients, showed that ctDNA detection is more frequent in patients with more advanced cancer (ctDNA was detected before treatment in 24%, 77%, and 87% of patients with stages I, II, and III of the disease, respectively) [32].

### 2.2. cfDNA and ctDNA Differentiation

Nowadays, it is impossible to differentiate ctDNA from normal cfDNA during nucleic acid isolation. This is because the similar lengths of both ctDNA and cfDNA are extracted under the same conditions. The most common approach to discriminate those nucleic acid types is to analyze tumor-specific mutations or methylation patterns within the extracted material [17,18]. It is also possible to focus on quantitative changes. For instance, deleted regions in tumor cells’ genomes should be underrepresented in ctDNA. Moreover, overrepresented sequences amplified in tumor cells may be observed at the copy number alteration (CNA) level in many patients with cancer [15]. However, to differentiate ctDNA from cfDNA at the molecular level, a proper assay must be selected; thus, knowledge about the type of cancer is indispensable [14].

Tumor-specific mutations and rearrangements in ctDNA may be analyzed during whole genome sequencing (WGS) or targeted sequencing. There are few assays targeted at specific patient cohorts that have been approved by the FDA (Food and Drug Administration) [18,33,34]. Currently, these tests apply for the assessment of prognosis and qualification for treatment but not for cancer screening [18,33]. During genotyping and distinguishing ctDNA from normal cfDNA, it is important to take into consideration the clonal hematopoiesis phenomenon, which stands behind the presence of somatic mutations in hematopoietic cells, as well as the fact that peripheral blood cells (PBCs) are a significant source of cfDNA in the bloodstream. Not considering clonal hematopoiesis in genotyping may lead to falsely categorizing white blood cell mutations as tumor-specific mutations. It is especially important in elderly patients in which clonal hematopoiesis occurs more often due to normal apoptotic processes [23,35]. One of the possibilities to eliminate false positive genotyping in liquid biopsies is a comparison of results between the sequencing of ctDNA and genomic DNA (gDNA) of tumor biopsy delivered from the same patient. This is viable due to approximately 60–80% mutation compliance in ctDNA and DNA from cancer cells of various cancers. The second option to distinguish ctDNA from normal cfDNA is a comparison of the mutation landscape between cfDNA and gDNA isolated from white blood cells, considered the normal match, which delivers a large repertoire of clonal hematopoietic alterations [18,36,37].

On the other hand, an analysis of methylation can be used to identify the kinds of cells that release cfDNA into the bloodstream and may become a powerful tool in distinguishing cfDNA from ctDNA. The discrimination of DNA released from “healthy” and tumor cells may be helpful in spotting tumor origin [38,39]. This kind of differentiation of normal cfDNA and ctDNA is possible due to the variety of methylation patterns in normal and cancer cells. In particular, the increased methylation of suppressor genes may be one of the first noticeable changes during tumorigenesis. Due to this fact, an analysis of CpG island (CGI) methylation seems to be a promising biomarker of neoplastic processes in various cancer types. Numerous DNA methylation biomarkers are already known and collected in The Cancer Genome Atlas (TCGA; http://cancergenome.nih.gov (accessed on 27 December 2023)), but it is necessary to increase their sensitivity and specificity before they can be widely clinically used. Nevertheless, several tests based on the analysis of suppressor gene methylation in liquid biopsies (e.g., peripheral blood and bronchoalveolar lavage) have been approved for in vitro diagnosis (IVD) of various cancers (e.g., colorectal cancer and NSCLC). Moreover, it has been considered that methylation haplotyping of ctDNA may be useful for early tumor detection, an assessment of progression, and the confirmation of the metastases’ presence [40,41,42].

In the ctDNA of cancer patients, it is also possible to quantitatively detect large changes, such as chromosomal rearrangements and CNAs. Research conducted by Jiang et al. on hepatocellular carcinoma patients showed that the size of chromosomal arms (with and without deletions) in tumor tissue reflected the size in ctDNA. If the chromosomal arm was amplified in cancer cells, its contribution to plasma DNA was proportionally increased, and when it was deleted, its contribution was decreased [43]. Sivapalan et al. focused on, among others, plasma aneuploidy in small cell lung cancer (SCLC) patients as well. During their contributed research, they found plasma aneuploidy of specific chromosomes in ctDNA, and the aberrations detected in ctDNA were reflected in changes in tumor tissue. However, to differentiate tumor-derived aberrations from biological noise and changes related to clonal hematopoietic, a highly optimized NGS assay and computational error correction were applied [44].

Calculations relying on the dependencies described above are being used in some studies. The Tumor Fraction Estimator (TFE) is based on the measurement of tumor aneuploidy. In the TFE, the calculation of the ctDNA fraction is possible due to the comparison of tested sample sequencing with a set of samples with well-known tumor fractions [45]. The second algorithm, maximum somatic allele frequency (MSAF), is based on the measurement of the maximum somatic allele frequency of somatic and likely somatic mutations in ctDNA. MSAF considered that those frequencies’ participation correspond to the abundance of ctDNA in cfDNA. Unfortunately, MSAF does not consider clonal hematopoiesis; thus, in many cases, TFE is more often recommended [45,46]. On the other hand, Schrock et al. observed in NSCLC patients that a lower MSAF level was associated with a higher risk of missing important genomic alterations in the plasma, such as exon 19 deletion and p.Thr790Met substitution in the *EGFR* gene [47]. Moreover, in a B-F1SRT study, an MSAF of <1% was associated with a higher response rate and better PFS during atezolizumab therapy in advanced NSCLC, but this effect was dependent on baseline tumor mutation burden (TMB) [48,49]. Gandara DR et al. suggested that a low MSAF could contribute to a poorer consistency between TMB in the bloodstream (bTMB) and tumor (tTMB) [50]. Incorporating MSAF with bTMB can partially improve the differentiation between patients with or without survival benefits from immune checkpoint inhibitors (ICIs). MSAF alone or in combination with bTMB can effectively distinguish NSCLC patients with or without OS (overall survival) and PFS (progression-free survival) benefit from atezolizumab compared with docetaxel. MSAF and the combined bTMB-MSAF classification may become practical, non-invasive biomarkers for atezolizumab efficacy in advanced NSCLC patients [49]. However, MSAF clinical value requires confirmation in a larger cohort within a standardized clinical trial.

## 3. Variant Allele Frequency (VAF)

**Figure 2:**
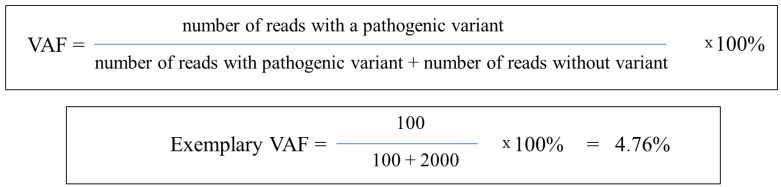
Method of VAF calculation in cancer patients using NGS technology.

The breakthrough in ultra-deep and sensitive metrics for mutation analysis in cancer patients has provided a modern approach to ctDNA analysis that focuses on variant allele frequency (VAF) [51]. However, in the literature, VAF is often substituted by mutant allele frequency (MAF), which carries the same meaning. However, the term MAF may also be used as minor allele frequency and refers to the frequency of germline alleles in large cohorts analyzed by genome-wide association studies (GWASs) [52]. For this reason, a good practice is to use VAF as a metric of cancerous variants. VAF is a metric characterized as the measurement of the specific variant allele proportion within a genomic locus determined by both NGS- and PCR-based methods (Figure 2) [17,53]. The prognostic and predictive roles of VAF have been evaluated across different studies [12,54,55,56,57]. However, any validated VAF thresholds may increase its clinical utility. This may be due to the limited standardization between diagnostic assays used in variant detection [53]. On the other hand, VAF provides insights into tumor clonality in somatic genomic testing, yielding a strong rationale for targeting dominant cancer cell populations that may pave the way for a new decision-making tool for targeted therapy selection [58].

VAF refers to a fraction of alleles carrying a specific genomic alteration [13]. A high VAF value suggests that a high percentage of tumor cells carry a particular genomic alteration. In such situations, targeted therapy may be easier to select and more effective [17,53]. Conversely, low VAF values suggest the low clonality of the genomic alteration [59,60]; thus, targeted therapy may be less effective because of the presence of other subclones carrying distinct genomic profiles [53,61]. Moreover, VAF may allow distinguishing driver mutations from passenger mutations since driver mutations refer to genomic alterations that directly contribute to the development and progression of cancer [62,63]. In this situation, high VAF occurs in genes that confer a selective clonal selection of cancer cells [53]. By contrast, passenger mutations represent genetic alterations that occur randomly in cancer cells, resulting from genetic instability. For decades it was considered that passenger mutations do not directly contribute to the progression of cancer [53,59]; however, previous comprehensive pan-cancer studies have indicated that passenger mutations may have important functional roles in driving cancer, as well as playing roles in tumor progression [64,65]. Moreover, passenger mutation analysis may allow an accurate classification of human tumors [66]. On the other hand, passenger mutations are not so actively promoted or enriched in the cancer cell population as drivers [63]; however, an analysis of their VAF may allow the measurement of the fitness of cancer cells, as well as the anti-tumor effects of chemotherapies [67,68].

Both metrics obtained from PCR- and NGS-based methods enable VAF calculation. VAF analyses based on PCR, e.g., digital PCR-based method (ddPCR) and Beads, Emulsion, Amplification, and Magnetics (BEAMing), provide high sensitivity and are more cost-effective than VAF calculated by NGS results [69,70]. However, NGS-based methods (such as whole exome sequencing, whole genome sequencing, or targeted sequencing) enable better genome coverage and the selection of the appropriate sequencing depth, allowing for the distinction of sequencing artifacts, as well as germline and somatic mutations [53].

One of the most concerning issues of VAF usage in liquid biopsy is a low concentration of ctDNA carrying the driver mutations in plasma. This limitation may affect the sensitivity of the detection method and lead to false negative results. Potentially, this problem can be eliminated by using larger plasma volumes for ctDNA extraction [53,71]. However, the ctDNA yield depends on many factors, starting with the pre-analytic conditions and chosen isolation assay, through to the type and stage of the tumor, and ending with the patient’s age. Moreover, cancer progression will also affect the ctDNA/cfDNA ratio [45,72]. At the step of sample preparation, it is important to centrifuge blood as soon as possible after collection to lower the risk of DNA contamination caused by cell lysis [73]. Various protocols suggest proceeding with centrifugation at different temperatures and speed conditions, depending on laboratory equipment; however, in general, centrifugation for 10 min at 1000–2000× *g* using a refrigerated centrifuge allows obtaining high-quality plasma, while centrifugation for 15 min at 2000× *g* removes platelets from the plasma [74]. Some protocols consider two cycles of centrifugation: the first cycle is performed at a lower speed to separate the plasma from morphotic elements, and the second one, at a higher speed, enables the reduction of cellular debris [75,76]. In the end, the prepared plasma can proceed immediately for further laboratory steps or be stored at −20 °C. Freezing at −80 °C is also acceptable, especially if long-term storage of the material is planned. However, it is recommended to avoid thawing and refreezing of plasma, which decreases its quality [76,77,78]. Although, in contrast to the isolation of DNA from FFPE (formalin-fixed paraffin-embedded) tissue, the usage of ctDNA does not involve formaldehyde contamination that may provide relatively fewer artifacts or unspecific results in analysis. Due to this, deamination does not affect ctDNA, and the risk of false positive results is lower in liquid biopsy [79]. Furthermore, single tissue collection by biopsy is related to a higher risk of tumor heterogeneity-related biases, while ctDNA is shed by all apoptotic or necrotic tumor cells, providing a representative material of the whole cancerous process for comprehensive genetic analysis [80,81,82].

### Usage of ctDNA VAF Analysis

The potential clinical application of VAF is related to the possibility of prognosis assessment or treatment response monitoring. It is considered that a high VAF rate in blood correlates with a large tumor volume [83,84], and especially with TMB, it may correlate with a worse prognosis [53]. Research conducted on different groups of patients showed an inversely proportional relationship between VAF and overall survival. This correlation occurred regardless of tumor type, which suggests the relevance of pre-treatment VAF analysis in the prognosis assessment of various patient groups. Moreover, it has been indicated that a higher VAF may be associated with shorter PFS regardless of therapy type [55,85,86].

Assessing predictive value and utility in the therapy selection of VAF is a consideration of interest to researchers. Different studies have indicated that a decrease in VAF correlates with response to therapy. Analogously, an increase in VAF may result from disease progression or recurrence. Moreover, VAF change could be noticeable earlier when compared to the radiographic or clinical evidence of response. It is also considered that VAF analysis can be used during assessment if adjuvant therapy would be beneficial and help to determine optimal treatment management [14,87,88]. Also, the introduction of VAF into the analysis of copy number variants (CNVs) may provide the optimal strategy for calling somatic CNAs [89]. However, CNV analysis alone seems to have higher predictive value, while VAF alone may be valuable in monitoring response to treatment. This way, CNV and VAF may complement each other during therapy decision making in certain groups of patients [90].

## 4. Assessment of Therapy Effectiveness in NSCLC Patients Based on VAF

Research performed on NSCLC patients indicated the potential clinical utility of VAF as a marker of response to various types of treatment. Most studies have focused on monitoring the dynamics of changes in mean or maximum VAF detected in ctDNA [91,92,93]. At this time, information about specific variants that are useful in assessing the effectiveness of therapy is limited. This issue seems to be of great interest to researchers, and the results are promising [91,93]. Firstly, VAF dynamics changes in patients treated with adjuvant chemotherapy correlated with radiological recurrence. In addition, it was suggested that VAF analysis may correlate with the risk of MRD and could potentially be used in the real-time monitoring of recurrence in patients after resection [7]. Unfortunately, low VAF values in patients with MRD may not be sufficient to reach the detection limit of currently used methods [37,81,94].

In a study performed on a group of 22 advanced NSCLC patients treated with immunotherapy (the anti-PD-1 antibody camrelizumab) or immunotherapy combined with anti-angiogenic therapy (the VEGFR2 inhibitor apatinib), VAF seemed to have potential as a marker of tumor progression or relief [95]. Studies have shown that a VAF decrease during therapy corresponds with a reduction in tumor size [14,95]. In 1 patient, out of 22, the tumor maximal diameter did not change after the treatment with combined immunotherapy, while VAF decreased. After initial stabilization, this patient experienced partial remission (PR). This case suggests that the sensitivity of VAF changes may be higher when compared to the radiographic assessment [95]. Similarly, Raja et al. also observed a change in the frequency of variants before a radiological response to durvalumab (anti-PD-L1 antibody) therapy in 42% of patients with bladder or non-small cell lung cancer [14]. Chen et al. have suggested that monitoring only a few mutations in ctDNA may be sufficient in treatment response prediction [14,95].

In the third phase of the CameL-sq trial, which included advanced squamous lung cancer patients treated with camrelizumab in combination with platinum-based chemotherapy plus, VAF measurements after two cycles of therapy were useful in discriminating patients with PR from patients with stable disease (SD) or progressive disease (PD). Furthermore, VAF assessment, at this time, showed a stronger correlation with response to therapy compared to ctDNA concentration analysis at the beginning of treatment or after two cycles [96]. Thompson et al. analyzed NSCLC patients treated with pembrolizumab (anti-PD-1 antibody) in monotherapy or in combination with chemotherapy as a first or second line of treatment. The molecular response was assessed as the ratio of mean ctDNA VAF during treatment to the mean ctDNA VAF at the baseline for *TP53*, *KRAS*, and *STK11* genes. It was reported that patients with response to therapy had significantly lower VAF (<50%) in the 6th week of therapy, which was followed by longer PFS, OS, and duration of treatment with pembrolizumab. Mutations in *TP53* and *KRAS* genes occurred with the highest frequency, while the presence of mutations in the *STK11* gene, considered as associated with worse outcomes in patients treated with pembrolizumab, was also found in several patients [92].

A meta-analysis conducted by Vega et al. included five studies on NSCLC patients treated with ICIs in monotherapy or in combination with other agents. It was indicated that mean and maximum VAF values were strongly associated with outcomes, while median VAF gave inconsistent results. Moreover, the authors did not observe a correlation between baseline VAF and outcome, although they did not exclude the possibility of this value in clinical usage [91].

The IMPower 010 study assessed the presence of ctDNA in the blood serum of patients after surgery and after adjuvant chemotherapy before adjuvant atezolizumab (an anti-PD-L1 antibody) immunotherapy or best supportive care (BSC). The first analysis indicated that the use of adjuvant chemotherapy led to a decrease in ctDNA concentrations in blood sera in 62% of initially ctDNA-positive patients, which was then associated with a longer time to disease recurrence in the group of patients receiving BSC. It was shown that atezolizumab, when compared to BSC, reduced the risk of disease recurrence by 30% in the group of patients with decreased ctDNA concentration observed after adjuvant chemotherapy. However, no such significant differences occurred in the group of patients without a decrease in ctDNA concentration after chemotherapy. It also appears that in the group of patients with a negative ctDNA result after chemotherapy, atezolizumab delayed the conversion of patients to a positive ctDNA result [97].

Likewise, with regard to the research on ICIs mentioned previously, studies performed in NSCLC patients treated with ALK tyrosine kinase inhibitors (ALK-TKIs) and EGFR tyrosine kinase inhibitors (EGFR-TKIs) indicated the potential application of ctDNA and VAF analysis in therapy effectiveness prediction. Soo et al. investigated VAF for fusion or single nucleotide variants (SNVs) of the *ALK* gene in patients who received lorlatinib or crizotinib (CROWN clinical trial). Different somatic alternations, including somatic variants of the ALK gene, were analyzed. The VAF change (delta VAF (dVAF)) between the baseline value and the VAF measured between the 4th and 24th week of treatment was measured. A dVAF ≤ 0, both for *ALK* gene alternations and any other somatic alternations, in the lorlatinib-treated group, was associated with longer PFS and tumor size decrease. However, this correlation was observed for VAF measured at week 4, but it was not confirmed for VAF analyzed at week 24. This may suggest the usage of VAF of the *ALK* gene as a marker of early response [98].

In their case study, Begum et al. demonstrated that VAF analysis in ctDNA can be a tool for the detection and monitoring of response or resistance to treatment with crizotinib and lorlatinib in NSCLC patients with *ROS1* gene rearrangement. The acquisition of mutations, c.G2101A, in the *ROS1* gene and changes in VAF detected by NGS indicated a moment of progression during crizotinib treatment in this patient. The emphasis was mostly on acquired G2101A substitution in the *ROS1* gene, which seemed to be associated not only with resistance to crizotinib but also with sensitivity to lorlatinib; thus, it could guide therapy decision making. Regrettably, extended research and validation of the results on a larger cohort are still necessary [99].

The application of ctDNA VAF analysis was also examined on a group of NSCLC patients with *EGFR* gene mutations treated with osimertinib, the third generation of EGFR-TKI. It was observed that patients included in the group of non-responders had higher VAF values of specific, actionable *EGFR* mutations in comparison to responders (patients with PR or SD). The research suggested that tracking VAF dynamics in ctDNA may have utility in monitoring response to EGFR-TKI [100].

Vaclova et al. assessed the VAF of p.Thr790Met mutations in the *EGFR* gene in patients treated with EGFR-TKI and proposed 30% VAF as a cut-off value to differentiate patients with clonal (VAF ≥ 30%) and subclonal (VAF < 30%) p.Thr790Met mutations. However, regardless of the clonality of p.Thr790Met substitution, both groups benefited from the therapy with osimertinib. The follow-up indicated that after 3 weeks of treatment, the VAF value decreased, and after 6 weeks, it either decreased or the value remained at a very low level (VAF < 1%). These observations were independent of clonality status, but were more variable in the subclonal group [101]. Contrary to the aforementioned studies, Ai et al. did not observe a correlation between the ctDNA VAF of the *EGFR* gene and the effectiveness of EGFR-TKI treatment. However, their study indicated that clonal dominance of *EGFR* gene mutations is an independent factor associated with the efficiency of EGFR-TKI treatment in patients with advanced NSCLC [102]. Furthermore, many current clinical trials aim to evaluate the VAF clinical value in larger NSCLC patient groups (Table 1).

## 5. Technical Aspects of VAF Evaluation in NSCLC Patients

### 5.1. Biological Factors Affecting VAF Measurement

During the analysis of VAF results, it is also important to take into account the possible influence of other factors on VAF value. For instance, it was shown that tumors with a high copy number of genes, especially amplification, may have significantly higher VAF values [103,104]. Nevertheless, it is being considered that the use of parameters such as ΔVAF can be valuable in the evaluation of ctDNA changes in patients with low or undetectable copy number variants and single nucleotide variants [105,106]. ctDNA concentration and VAF seem to correlate with tumor size; however, due to the detection limit of currently used platforms, VAF assessment appears to be the most optimal in patients with a tumor volume of at least 10 cm^3^, while a smaller tumor volume might release a borderline amount of ctDNA for sensitive detection [107].

On the other hand, VAF and tumor size may also be dependent on occurring mutations. A stronger correlation between VAF and tumor size appears in *KRAS*- and *TP53*-mutated tumors compared to *EGFR*-mutated tumors. Contrarily, NSCLC tumors with *TP53* or *EGFR* mutations are most likely to shed ctDNA [103]. Moreover, the VAF value may vary in NSCLC patients depending on the presence and localization of metastatic lesions. Belloum et al. indicated that patients with oligo-brain metastases had lower VAF values compared to patients in whom metastases occurred not only in the brain but also apart from the central nervous system [108]. However, the highest VAF values were observed in NSCLC patients with metastases in locations other than the brain, especially metastases with high vascularity in the kidneys, adrenal glands, liver, or spleen [103]. This discrepancy may result from the presence of blood–brain barriers that hinder the release of tumor cells and their products, such as ctDNA, into the bloodstream [108].

### 5.2. Appropriate Methodology Selection

Choosing the optimal tool to calculate VAF seems to be an issue. Research by Cheng et al. indicated that tumor-informed assay (i.e., a personalized assay based on prior tissue genotyping) enabled the detection of VAF, rather than tumor-agnostic assay (i.e., an assay independent of tumor profiling), and frequently presented negative results. The results suggested the applicability of personalized ctDNA tumor-informed assay in the monitoring of patients treated with ICIs in monotherapy or combination with chemotherapy in cases when tissue is not available for examination [109,110,111]. Furthermore, in patients where low VAF values are expected, it may be beneficial to choose the ddPCR technique rather than NGS due to the higher sensitivity of ddPCR. However, it is important to consider that ddPCR enables the detection of fewer mutations than NGS approaches. Further validation of methods based on NGS could improve its sensitivity and expand the possibility of VAF analysis in clinical practice [100,112,113].

Finally, it is also important to remember that ctDNA may be extracted from different body fluids apart from peripheral blood. In NSCLC patients, bronchoalveolar lavage (BAL) or bronchial washing (BW) fluid could be analyzed. The concordance of driver mutations present in BAL or BW fluids with tumor tissue can reach 95% [114]. In the BAL, VAF values and ctDNA concentration are higher when compared to plasma; therefore, more tumor-driver mutations can be detected. As a result, a BAL assessment may be especially useful when blood samples have a low yield of ctDNA. Due to these factors, it is even considered that BAL or BW fluid analyses may be useful in lung cancer diagnosis, especially in patients with non-diagnostic biopsies. A crucial disadvantage of those materials’ analyses is the necessity of performing bronchoscopy, which is an invasive procedure. However, bronchoscopy is often performed in patients with suspected NSCLC due to existing medical indications; therefore, material could be collected during standard procedures [114,115,116]. In patients with metastases in the central nervous system, cerebrospinal fluid (CSF) appears to be another useful source of ctDNA for VAF analysis. One of the most important possibilities of its usage seems to be facilitating the selection of therapy in patients with intracranial progression. However, lumbar puncture is an invasive procedure, and the possibility of regular monitoring of CSF ctDNA seems to be limited in metastatic NSCLC patients [108,117].

## 6. Conclusions

VAF seems to be a promising new tool in therapy effectiveness monitoring, but some key issues need further investigation before its potential implementation for clinical use. The first issue is the necessity of expanding and standardizing the method of VAF analysis. In particular, several or single genetic alterations should be identified for each treatment regimen as the measurable parameters for VAF tracking during clinical follow-up. In NSCLC patients, especially those treated with immunotherapy, the calculation of VAF and its monitoring in different studies has been based on various variants in several genes detected in a particular person. Even though it is possible to design a customized gene panel, it may be difficult to develop it optimally and then put it into widespread use due to the absence of fully consistent research results. Further investigation is also required to determine the optimal frequency of blood collection for VAF assessment. Another crucial matter is the development of the optimal calculation of VAF metrics—different studies are based on mean, median, or highest VAF, and values are calculated using different formulas. It should also be indicated whether it is preferable to calculate VAF once or multiple times, or concerning some baseline value, and how often VAF analysis should be performed.

The second limitation to the use of VAF testing is the methodological aspect. Many of the currently used methods are insufficient for assessing the effectiveness of therapy, especially MRD monitoring, due to the relatively low sensitivity or the limited number of mutations that can be detected. Further developments in sequencing and reductions in the costs of currently used methods could not only enable the expansion of research but also open the possibility of the clinical usage of ctDNA VAF analysis. Before implementation in clinical routine, methodologies need to be validated, including developing appropriate sensitivity and specificity. The presence of false negative and false positive results should be as low as possible.

In conclusion, despite the mentioned difficulties, VAF analysis seems to have great potential in the different aspects of cancer patients’ diagnosis, especially due to the non-invasive procedure of ctDNA collection. Ongoing clinical trials and further sequencing methodology development, especially on single-cell resolution, may fill the gap in the current knowledge, paving the way for early cancer detection, cancer interception, and MRD monitoring, as well as measuring the treatment effect or tracking the metastatic spread at the molecular level by VAF analysis.

## Figures and Tables

**Table 1:** A summary of clinical trials that are already recruiting NSCLC patients to evaluate VAF metrics for monitoring the effectiveness of personalized treatment. Data were collected from the ClinalTrials.gov database (http://clinicaltrials.gov/ (accessed on 4 January 2024)).

Clinical Trials ID (Duration)	Title	Main Location (Sponsor)	Number of Participants (Trial Type)	Primary Outcomes
NCT05708599 (02.2023–02.2028)	A Study to Compare Tissue and Liquid Biopsies in People With Different Types of Cancer	Germany (Boehringer Ingelheim)	180 (Interventional)	The mean VAF of mutations in ctDNA samples over the timescale of the patient’s treatment course
NCT05429320 (06.2022–06.2025	A Study of Local Ablative Therapy (LAT) in People With Non-Small Cell Lung Cancer (NSCLC)	USA (Memorial Sloan Kettering Cancer Center)	117 (Interventional)	Measure the reduction in mean VAF by 6 months after local ablative therapy
NCT05921474 (04.2023–12.2023)	Detection of Circulating Tumor DNA After Stereotactic Ablative Radiotherapy in Patients With Unbiopsied Lung Tumors (SABR-DETECT)	Canada (Lawson Health Research Institute)	100 (Observational)	Increases in VAF or quantifiable ctDNA from baseline to post-treatment samples (in patients with detectable ctDNA at baseline)
NCT05221372 (02.2017–01.2031)	ProSpecTive sAmpling in dRiver muTation Pulmonary Oncology Patients on Tyrosine Kinase Inhibitors (START-TKI)	Netherlands (Erasmus Medical Center)	1300 (Observational)	The relative presence of primary mutations and resistance mutations in plasma levels under the treatment of a small molecule kinase inhibitor until the progression of disease measured in VAF
NCT04122833 (09.2019–12.2024)	Impact of Concomitant Genetic Alterations in *EGFR* Mutated Adenocarcinoma by NGS Analysis: A Multicenter Study	South Korea (Konkuk University Medical Center)	80 (Observational)	The correlation between the change in VAF and drug response in matched tumor tissues before and after TKI treatment
NCT05102110 (12.2021–12/2023)	Feasibility Study to Investigate Rectal Mucus in Aero-Digestive Tract Cancer (ORI-EGI-03)	United Kingdom (Origin Sciences)	300 (Observational)	The correlation of SNP allele frequency in genes associated with known aero-digestive cancers in paired samples of tumour type and rectal mucus
NCT05254795 (04.2022–12.2036)	Precision Medicine Randomized Clinical Trial Comparing Molecular Tumor Board Assisted Care to Usual Care (PRiMAL)	USA (Jill M Kolesar)	500 (Interventional)	The association of ctDNA VAF with 1-year overall survival
NCT05782361 (05.2023–02.2028)	POTENT-Tepotinib in Combination With Pembrolizumab in NSCLC	United Kingdom (Institute of Cancer Research)	38 (Interventional)	The determination of allele frequency of genomic aberrations including, but not limited to, the *MET*, *EGFR*, *BRAF*, and *KRAS* genes in plasma
NCT03778229 (01.2019–05.2025)	Osimertinib Plus Savolitinib in EGFRm+/ΔMET+ NSCLC Following Prior Osimertinib (SAVANNAH)	USA (AstraZeneca)	360 (Interventional)	Total clearance of *EGFR* mutations at 6 weeks after osimertinib and savolitinib therapy initiation (the percentage and absolute change from baseline in *EGFR* mutation allele frequencies)

## Abbreviations

ACT	Adjuvant chemotherapy
ALK-TKI	ALK–tyrosine kinase inhibitor
BAL	Bronchoalveolar lavage
BSC	Best supportive care
BW	Bronchial washing
cfDNA	Circulating free DNA
ctDNA	Circulating tumor DNA
CNA	Copy number alteration
CNV	Copy number variation
CSF	Cerebrospinal fluid
EGFR-TKI	EGFR–tyrosine kinase inhibitor
FFPE	Formalin-fixed paraffin-embedded
ICI	Immune checkpoint inhibitor
LB	Liquid biopsy
MAF	Mutant allele frequency
MRD	Minimal residual disease
MSAF	Maximum somatic allele frequency
NET	Neutrophil extracellular trap
NSCLC	Non-small cell lung cancer
OS	Overall survival
PD	Progressive disease
PFS	Progression-free survival
PR	Partial response
SD	Stable disease
SNV	Single nucleotide variant
TCGA	The Cancer Genome Atlas
TFE	Tumor Fraction Estimator
VAF	Variant allele frequency
WES	Whole exome sequencing
WGS	Whole genome sequencing
